# The influence of suturectomy on age-related changes in cerebral blood flow in rabbits with familial bicoronal suture craniosynostosis: A quantitative analysis

**DOI:** 10.1371/journal.pone.0197296

**Published:** 2018-06-01

**Authors:** Ramesh Grandhi, Geoffrey W. Peitz, Lesley M. Foley, Christopher M. Bonfield, Wendy Fellows-Mayle, T. Kevin Hitchens, Mark P. Mooney

**Affiliations:** 1 Department of Neurosurgery, University of Texas Health Science Center at San Antonio, San Antonio, TX, United States of America; 2 Pittsburgh NMR Center for Biomedical Research, Carnegie Mellon University, Pittsburgh, PA, United States of America; 3 High Field Animal Imaging Center, University of Pittsburgh, Pittsburgh, PA, United States of America; 4 Department of Neurosurgery, Vanderbilt University Medical Center, Nashville, TN, United States of America; 5 Department of Neurological Surgery, University of Pittsburgh Medical Center, Pittsburgh, PA, United States of America; 6 Departments of Oral Biology, University of Pittsburgh, Pittsburgh, PA, United States of America; 7 Department of Anthropology, University of Pittsburgh, Pittsburgh, PA, United States of America; 8 Deparment of Plastic Surgery, University of Pittsburgh, Pittsburgh, PA, United States of America; 9 Deparment of Orthodontics, University of Pittsburgh, Pittsburgh, PA, United States of America; 10 Department of Communication Science and Disorders, University of Pittsburgh, Pittsburgh, PA, United States of America; Medical University of South Carolina, UNITED STATES

## Abstract

**Background:**

Coronal suture synostosis is a condition which can have deleterious physical and cognitive sequelae in humans if not corrected. A well-established animal model has previously demonstrated disruptions in intracranial pressure and developmental abnormalities in rabbits with congenital craniosynostosis compared to wild type rabbits.

**Objective:**

The current study aimed to measure the cerebral blood flow (CBF) in developing rabbits with craniosynostosis who underwent suturectomy compared to those with no intervention and compared to wild type rabbits.

**Methods:**

Rabbits with early onset coronal suture synostosis were assigned to have suturectomy at 10 days of age (EOCS-SU, n = 15) or no intervention (EOCS, n = 18). A subset of each group was randomly selected for measurement at 10 days of age, 25 days of age, and 42 days of age. Wild type rabbits (WT, n = 18) were also randomly assigned to measurement at each time point as controls. Cerebral blood flow at the bilateral hemispheres, cortices, thalami, and superficial cortices was measured in each group using arterial spin-labeling MRI.

**Results:**

At 25 days of age, CBF at the superficial cortex was significantly higher in EOCS rabbits (192.6 ± 10.1 mL/100 mg/min on the left and 195 ± 9.5 mL/100 mg/min on the right) compared to WT rabbits (99.2 ± 29.1 mL/100 mg/min on the left and 96.2 ± 21.4 mL/100 mg/min on the right), but there was no significant difference in CBF between EOCS-SU (97.6 ± 11.3 mL/100 mg/min on the left and 99 ± 7.4 mL/100 mg/min on the right) and WT rabbits. By 42 days of age the CBF in EOCS rabbits was not significantly different than that of WT rabbits.

**Conclusion:**

Suturectomy eliminated the abnormally increased CBF at the superficial cortex seen in EOCS rabbits at 25 days of age. This finding contributes to the evidence that suturectomy limits abnormalities of ICP and CBF associated with craniosynostosis.

## Introduction

Craniosynostosis is a condition in which one or more of the calvarial sutures fuse prematurely. Though there are syndromic causes, most commonly, craniosynostosis is considered to be idiopathic, and its incidence is estimated at 1 out of 2500 live births. Involvement of the coronal sutures (CS) constitutes approximately 25% of cases [1]. Children with uncorrected craniosynostosis develop abnormal head shapes, based on the particular suture that has prematurely closed. The restriction imposed by the closure of the suture involved may not allow for the developing brain to have enough space for the growth. Left untreated, the downstream clinical sequelae may include visual impairment, headaches, and delays in cognitive and psychomotor development [2–3] with consequent impairment of mental development and significant reductions in IQ [4]. The causes of these sequelae are multifactorial, but intracranial hypertension may be an important component.

The prevalence of intracranial hypertension in patients with craniosynostosis is unclear due to variation in method, duration of measurement, and ambiguity on what threshold value of intracranial pressure (ICP) is considered abnormal in each age group. Whereas reported prevalence ranges from 30–40% in syndromic craniosynostosis and 15–20% in nonsyndromic craniosynostosis, Tamburrini et al. (2005) argued for prolonged monitoring of ICP and attention to plateau waves and determined that 87.5% of syndromic patients with craniosynostosis had abnormal ICP [5–6]. While definitions of abnormal ICP vary, within-study comparisons between ICP before and after surgical correction of craniosynostosis have not shown a consistent normalization of ICP after surgery [5]. In addition, important factors besides decreased intracranial volume, such as airway obstruction, impaired venous drainage, and altered CSF flow and absorption, contribute to intracranial hypertension in craniosynostosis patients. It is unproven which, if any, of these factors may be responsible for reported neurocognitive outcomes [7, 8]. Therefore, besides the consequent changes in head shape achieved with surgical intervention, it is important to investigate other neurophysiologic phenomena to understand the potential ramifications of surgery in patients with craniosynostosis.

Along with increased intracranial pressure (ICP) [9–13], other aberrations in neurophysiologic parameters have been noted, most notably, abnormal cerebral blood flow (CBF) [14–16]. Observed downstream neuroanatomic consequences of these perturbations include white matter injury [17] and atypical venous drainage [18–20]. Examining CBF before and after surgical intervention for craniosynostosis could help characterize the effect of surgery on the pathophysiology of craniosynostosis.

Conventional anatomical magnetic resonance imaging (MRI) allows for detection of structural changes in the brain in a non-invasive manner, but only after potentially irreversible damage has been done. Anatomical MRI does not allow for the study of CBF. The use of arterial spin labeling MRI (ASL-MRI) to serially and non-invasively study CBF represents a crucial adjunctive imaging modality.

We have developed an inbred rabbit model of early-onset bilateral coronal craniosynostosis (EOCS) and have extensively described growth changes in the cranial vault and intracranial contents on these rabbits. Phenotypically, EOCS rabbits exhibit bony bridging of the coronal suture by 21 days gestation (gestation is 30 days in the rabbit), obliterated coronal sutures at birth, coronal ridging and brachycephalic cranial vaults by 10 days of age, and secondary changes in the cranial vault base, brain, and intracranial volume by 42 days of age [9, 10, 12, 13, 17, 21–24]. CBF was studied in EOCS rabbits at 10, 25 and 42 days of age using ASL-MRI. A previous study from our group revealed CBF to be similar between wild-type (WT) and EOCS subjects, with the exception of the superficial cortex surfaces in EOCS rabbits at 25 days of age [25]. A two-fold increase in superficial cortex CBF at 25 days of age was noted. However, by 42 days of age, the CBF reduced back to normal values. Interestingly, as noted by Fellows-Mayle and colleagues’ (2000) study, ICP values in EOCS rabbits mirror the increases in CBF seen: at 25 days of age, the EOCS subjects had significantly increased ICP, with a decrement noted by Day 42 [9]. Additionally, brain morphology studies in EOCS rabbits showed that significant brain atrophy occurred in these animals [21, 23, 24]. Given that brain growth accelerates in rabbits from early gestation to 30 days of age [23], which also corresponds to peak CBF and ICP levels seen at 25 days of age in EOCS rabbits, these studies suggest that perhaps cerebral autoregulation results in hyperemia at the pial surfaces in response to increased ICP; however, due to inadequate CBF, cerebral atrophy eventually occurs with consequent ICP reduction by day 42.

The present study was undertaken to serially map CBF in our animal model and determine the impact of suturectomy on CBF in EOCS subjects. We used ASL-MRI to show the age-related changes in CBF, comparing wild-type subjects, and EOCS subjects with and without suturectomy over various time points. In light of the previous studies showing age-related increased ICP and CBF in rabbits with craniosynostosis and the prevention of early increased ICP via suturectomy [9, 12, 24, 25], we investigated the hypothesis that early suturectomy in rabbits with bicoronal craniosynostosis will result in the abolishment of the age-related increase in CBF seen in the ipsilateral superficial cortical surfaces of our rabbit craniosynostosis model. By analyzing the differences in cerebral blood flow seen over time in subjects undergoing suturectomy versus those that do not undergo suturectomy, one may obtain clinically-relevant information and potentially determine whether a critical period exists during which suturectomy is indicated to prevent a cascade of changes in neurophysiologic parameters that may result in secondary brain injury.

## Materials and methods

### Animal model

This study used a breeding colony of New Zealand white rabbits with naturally occurring coronal suture synostosis [15, 21–25]. All rabbits were born and maintained in the vivarium at the Department of Anthropology, University of Pittsburgh and housed in stainless steel cages with access to food and water ad libitum. Rabbits were housed with mothers and littermates, up to the time of the experiment. Approval by the University of Pittsburgh Institutional Animal Care and Use Committee (#12100948) was obtained, and the experiment was reported in compliance with Animal Research: Reporting in Vivo Experiments guidelines [26].

**Fig 1:**
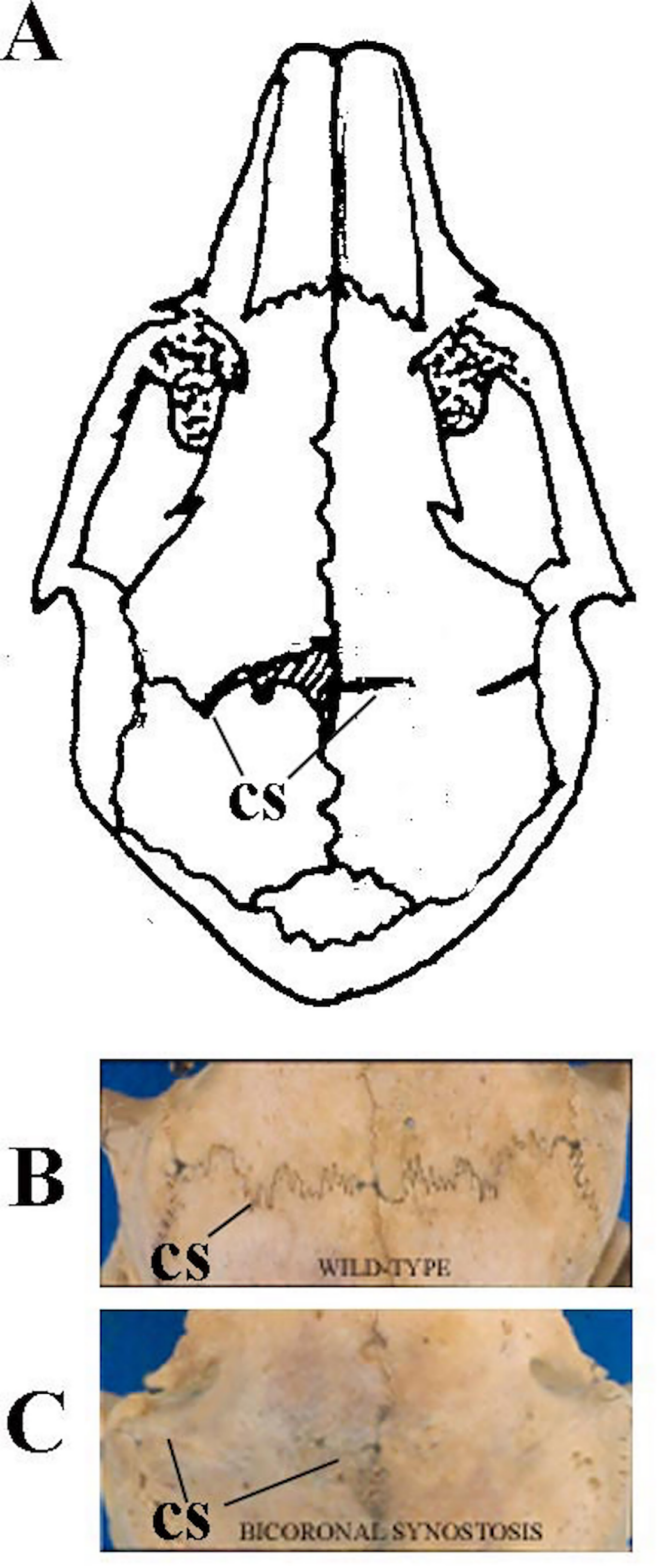
Representation of rabbit coronal sutures in cartoon (Panel A) and photographs (Panels B and C). Panel B demonstrates patent coronal sutures in a wild type rabbit, and Panel C demonstrates bilaterally fused coronal sutures in a rabbit with early-onset craniosynostosis (EOCS).

Synostotic rabbits were initially diagnosed at 10 days of age using previously described criteria [9, 10, 12, 13, 17, 21–24]. Briefly, all rabbits were anesthetized with an IM injection (0.59 mL/kg) of a solution containing 91% Ketaset (ketamine hydrochloride, 100 mg/mL, Aveco Co., Inc., Fort Dodge, IA) and 9% Rompun (xylazine, 20 mg/mL, Mobay Corp., Shawnee, KS). The scalps were then shaved, depilated, and prepared for surgery. Calvaria were exposed by midline incision of the scalp, and the skin was reflected laterally to the supraorbital borders. Coronal suture morphology was assessed by direct observation and the degree of bony bridging and coronal suture mobility were ascertained (Fig 1). Rabbits with bilaterally fused coronal sutures with no mobility were diagnosed with early-onset coronal suture synostosis (EOCS) and were placed in the study.

**Fig 2:**
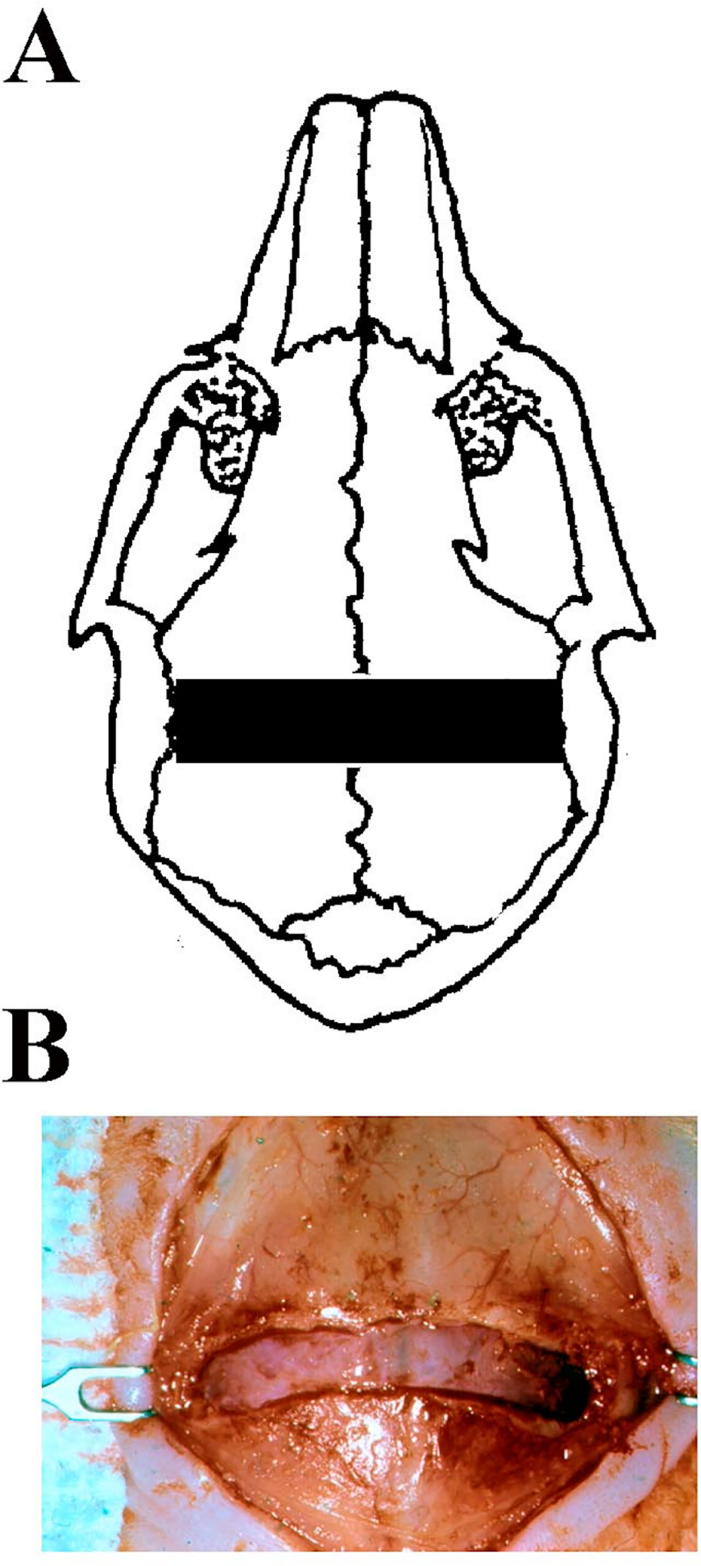
Cartoon (Panel A) and photograph (Panel B) of a rabbit skull after coronal suturectomy.

Removal of the coronal suture (EOCS-SU group) occurred immediately after diagnosis of synostosis in a random subset of EOCS rabbits. The coronal suture was removed by drilling 2mm anterior to, and 2mm posterior to the suture, for the length of the affected suture (Fig 2). A 4mm x approximately 20 mm piece of the skull was removed (Fig 2). The skin of the head was sutured and the rabbit was monitored during recovery. Rabbits were given enrofloxacin at a dose of 2 mg/kg twice a day subcutaneously and buprenorphine at a dose of 0.02 mg/kg subcutaneously immediately after surgery and for 2 days post operatively. The scalp incision was monitored daily and the skin sutures were removed at 20 days of age. At the conclusion of the experiment, rabbits were euthanized with Fatal Plus at a dose of 100 mg/kg intravenous.

Rabbits were randomly assigned into the following experimental groups: wild-type control rabbits (WT) at 10 days of age (n = 6); EOCS rabbits at 10 days of age (n = 6); EOCS-SU rabbits at 10 days of age (n = 5); WT rabbits at 25 days of age (n = 6); EOCS rabbits at 25 days of age (n = 6); EOCS-SU rabbits at 25 days of age (n = 5); WT rabbits at 42 days of age (n = 6); EOCS rabbits at 42 days of age (n = 6); and EOCS-SU rabbits at 42 days of age (n = 5).

### Protocol for MRI studies of CBF

For magnetic resonance studies, at or near 10, 25, and 42 days of age, rabbits were transported to the Pittsburgh NMR Center for Biomedical Research at Carnegie Mellon University. Rabbits were preanesthetized with an intramuscular injection of ketamine (100mg/ml) and xylazine (20 mg/ml) in a 9:1 solution at a dose of 0.6 mg/kg. All rabbits underwent tracheotomy and were ventilated (Harvard Apparatus, Holliston, MA) via an endotracheal tube with 1–1.5% isoflurane in a N_2_O:O_2_ (1:2) gas mixture. The rabbits were placed in dorsal recumbency and an indwelling catheter was inserted into the femoral artery. Arterial blood gases were collected immediately before and after MRI. Arterial CO_2_ tension (P_a_CO_2_) was maintained between 20 and 46 mmHg for the duration of the study. This has been reported as the normal P_a_CO_2_ for rabbits [27]. During MRI assessment animals were maintained at 36±0.5°C via a warm air heating system (SA Instruments, Stony Brook, NY).

### MRI image acquisition

MRI studies were performed on a 4.7-T, 40-cm bore Bruker AVANCE-AV system, equipped with a 12-cm-diameter-shielded gradient insert and a 72-mm volume RF coil for 10 and 25 days of age. At 42 days of age, half-PCOS RF coil was used. T2-weighted spin-echo images were used to verify the position of the perfusion slice, which was based on Figure 70 in the *Atlas of the Rabbit Brain and Spinal Cord* [28], and were acquired with the following parameters: field of view (FOV) = 6.4x6.4cm, 2-mm slice thickness, TR/TE = 2,000/90 ms, two averages, five slices and a 128x70 matrix interpolated to 128x128. Perfusion studies were performed using continuous arterial spin-labeling imaging technique (spin-echo, 128x70 matrix, TR = 200 ms, summation of three echoes, TE = 10, 20, and 30 ms and two averages) [29]. The labeling and control continuous RF pulse for the inversion plane was positioned ±2 cm from the perfusion detection plane. The spin-lattice relaxation time of tissue water (T_1obs_) was measured from a series of spin-echo images (TR = 8,000, 4,300, 2,300, 1,200, 650, 350, 185, and 100 ms, TE = 9 ms, two averages and a 128x70 matrix) [30]. Spin-labeling efficiency, α, was determined from intensities within the carotid arteries (gradient echo, 45^o^ flip angle, eight averages, TR/TE = 100/9.6 ms, 256x256 matrix and spin-labeling applied at ±6 mm) [31].

### Data analysis

All image processing was performed with the Bruker ParaVision 5.0 image analysis software. Pixel by pixel maps of (M_C_ − M_L_) x MC^−1^ were generated from the perfusion data. M_C_ and M_L_ are the magnetization intensities from the control image and labeled image, respectively. T_1obs_ maps were generated from the series of variable TR images by a three-parameter, non-linear fit. Regional CBF was then calculated according to Zhang et al [32]. CBF = λ^.^(T_1obs_x2α)^−1^ x (M_C_ − M_L_) x MC^−1^, where λ is the blood–brain partition coefficient of water, with a spatially constant value of 0.9 mL/g assumed [33], and α is the spin-labeling efficiency measured in the carotids. T2-weighted images were used to define regions of interest (ROIs) in the left and right hemisphere, which were copied onto the CBF maps where the mean CBF across the left and right hemispheres were calculated to see if there were large difference between animals. Smaller, more specific regions within each hemisphere including the cortex, thalamus, and the superficial cortex surfaces were also defined, guided by assignments from the rabbit brain atlas [28]. Mean CBF values were also calculated in these individual, smaller ROIs to determine if specific regions within the hemisphere may be significantly different between groups.

### Statistical analysis

Mean blood flow data were compared between groups at each age, and significant differences were assessed using one-way ANOVA on SPSS software (v. 13, SPSS Incorporated, Chicago, IL). Post-hoc group comparisons were made, and the Bonferroni correction was used for the multiple comparisons. All differences were considered significant if p < 0.05.

## Results

**Fig 3:**
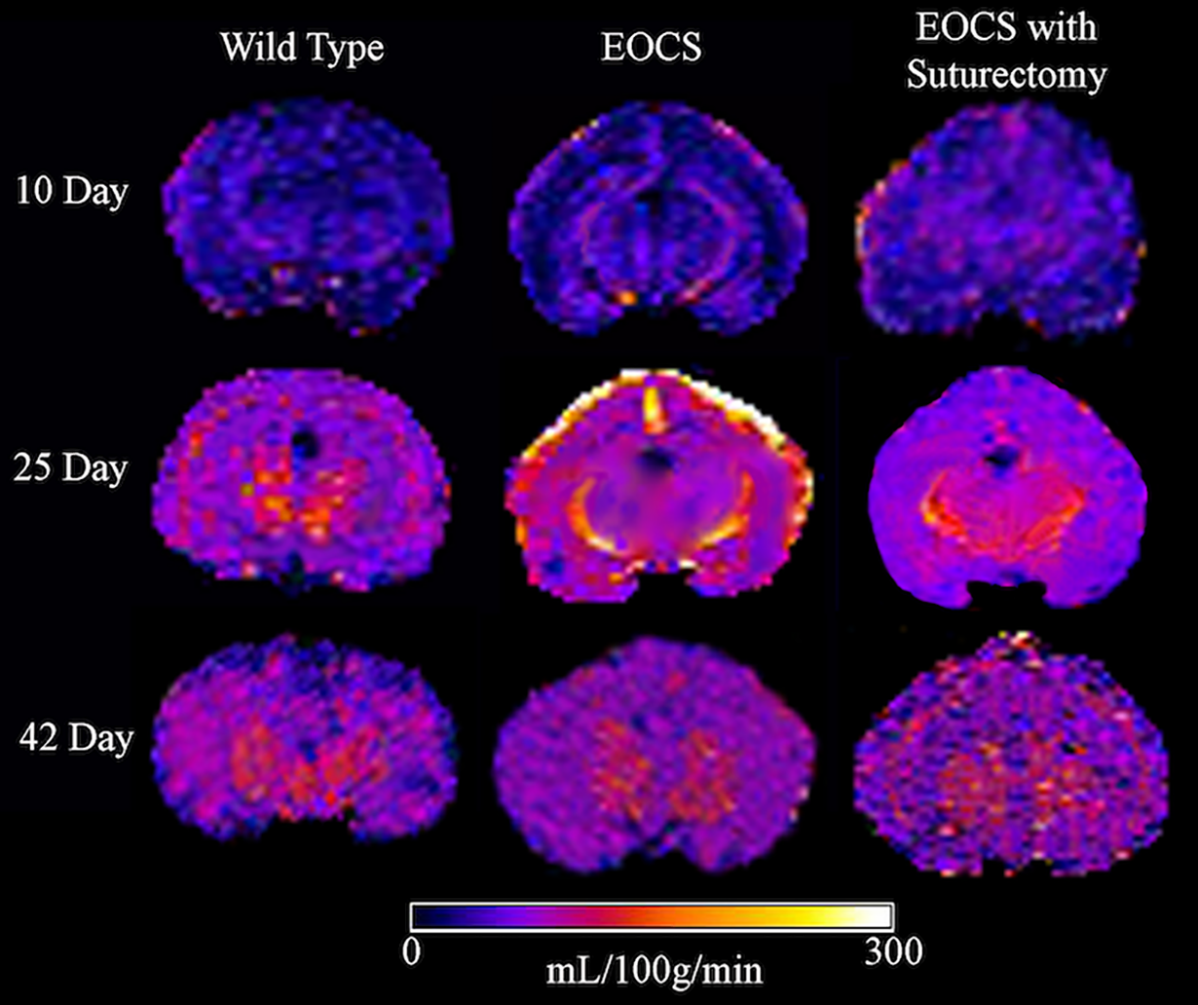
Representative cerebral blood flow maps from wild-type, EOCS, and EOCS with suturectomy rabbits at 10, 25, and 42 days of age.

Representative CBF maps for WT, EOCS, and EOCS-SU rabbits from the 10-day, 25-day, and 42-day groups are shown in Fig 3, and Table 1 shows mean CBF and associated standard deviation for each group. As shown in Table 1 and Fig 3, hemispheric, cortical, thalamic, and superficial cortical CBF was increased in the 25-day-old group and 42-day-old group compared to the 10-day-old group in WT, EOCS, and EOCS-SU rabbits. In most cases, this difference was significant at the 5% level. The exception was that mean CBF in the right cortex of the 42-day-old WT rabbits was not significantly different from that of the 10-day-old WT rabbits. Comparing WT and EOCS rabbits, the superficial cortical CBF in the 25-day-old EOCS (192.6 ± 10.1 mL/100 mg/min on the left and 195 ± 9.5 mL/100 mg/min on the right) rabbits was nearly double that of the of the 25-day-old WT rabbits (99.2 ± 29.1 mL/100 mg/min on the left and 96.2 ± 21.4 mL/100 mg/min on the right, *p* < .001), as shown in Fig 3. Superficial cortical CBF was not significantly elevated in EOCS-SU rabbits (97.6 ± 11.3 mL/100 mg/min on the left and 99 ± 7.4 mL/100 mg/min on the right) compared to WT rabbits at 25 days of age. By 42 days of age, superficial cortical CBF in EOCS rabbits was decreased to a level similar to the WT and EOCS-SU rabbits. In other ROIs, no significant differences in CBF were observed in WT compared to EOCS or EOCS-SU rabbits at any age.

**Table 1:** Regional cerebral blood flow by group.

	Left Hemisphere	Right Hemisphere	Left Cortex	Right Cortex	Left Thalamus	Right Thalamus	Left Superficial Cortex	Right Superficial Cortex
Wild-type								
10 days of age	35.7 ± 7.6	35 ± 7.6	45.7 ± 12.2	44.2 ± 11.8	36.7 ± 9.9	35.9 ± 9.1	41.7 ± 8.3	41.3 ± 5.7
25 days of age	90.7 ± 39.4 *	92.5 ± 39.4 *	90.2 ± 30.3 *	92 ± 33.4 *	122.3 ± 55.6 *	124.3 ± 58.3 *	99.2 ± 29.1 *	96.2 ± 21.4 *
42 days of age	72.8 ± 24.9 *	74.5 ± 21.3 *	78.3 ± 34.7 *	63.8 ± 22.8	99.3 ± 30.3 *	100 ± 36.4	84.3 ± 35.2 *	76.5 ± 23.9 *
EOCS								
10 days of age	29.5 ± 9.1	30.7 ± 7.2	32.7 ± 9.9	34.7 ± 8.2	36.2 ± 11.3	35.3 ± 9.6	38.7 ± 12.6	40.7 ± 12
25 days of age	92.2 ± 14.5 *	91.6 ± 15.6 *	108.8 ± 27.4 *	109 ± 29.5 *	129 ± 24.8 *	125.6 ± 23.3 *	192.6 ± 10.1 *, #	195 ± 9.5 *, #
42 days of age	70 ± 7.9 *	76 ± 8.9 *	70 ± 12 *	81.3 ± 6.1 *	112.5 ± 10 *	106.8 ± 14.2 *	81.5 ± 6.7 *	80.3 ± 5.5 *
EOCS-SU								
10 days of age	41 ± 14.5	44 ± 11.9	36 ± 6.8	43 ± 3.2	42.8 ± 11.6	40.2 ± 8.5	41.2 ± 8.6	47.4 ± 8.1
25 days of age	104.6 ± 6.9 *	106.4 ± 6.8 *	98.2 ± 5.5 *	105 ± 13.5 *	144.6 ± 11.2 *	140.4 ± 18.9 *	97.6 ± 11.3 *	99 ± 7.4 *
42 days of age	80.2 ± 7.4 *	74.5 ± 14.9 *	90.6 ± 9.1 *	79 ± 19 *	91.8 ± 11.1 *	83 ± 12.9 *	87.6 ± 13 *	76 ± 24.2 *

Data are mean ± standard deviation, units mL/100 mg/min. Data were analyzed with ANOVA tests, and results with p values < 0.05 were considered significant.

* Indicates significant difference from the corresponding 10-day-old group.

# Indicates significant difference from the wild-type group of the same age.

## Discussion

Our data demonstrates that in our rabbit model of early-onset craniosynostosis, age-related changes in CBF occurred in the superficial cortex compared to control animals and that this change was not seen in rabbits with early-onset craniosynostosis that underwent suturectomy. The increased CBF that is seen in the superficial cortex at 25 days coincides with the period of increased ICP previously reported by our group [9], which corroborates Auer et al.’s (1987) and Pennings et al.’s (2005) findings that during periods of intracranial hypertension and hypercapnia, respectively, owing to relaxation of smooth muscle within the arterial walls, pial arteries undergo compensatory dilation secondary to the process of cerebral autoregulation [34, 35]. By 42 days of age, CBF in our early-onset craniosynostotic rabbits decreased back to normal range and pial vasculature no longer demonstrated high CBF as compared to control animals, once again mirroring the age-related changes in ICP seen in craniosynostotic rabbits in which ICP decreases back to normal between 25 and 42 days of age [9].

Subjects that underwent suturectomy did not show a similar pattern of increased CBF at the superficial cortex at the 25-day time point. This study adds new evidence to the existing sequelae of craniosynostosis in the rabbit model, which includes decreased brain growth [23] and intracranial volume [24] and hyperactivity with early reflexive behavior in the early stages of life [36].

Based on the results of ASL-MRI, we found that cortical, hemispheric, and thalamic CBF were not different in rabbits with early-onset craniosynostosis of the coronal sutures when compared to age-matched wild-type rabbits and rabbits with early-onset craniosynostosis who underwent suturectomy. The increase in all groups seen between 10- and 25-days of age represents the normal pattern of CBF increase seen in the immature brain [37–39]; Tuor demonstrated low CBF in the early postnatal development in the rabbit (between 1 and 8 days of age) and a marked increase in CBF by 17 days of age [39]. One of the most important factors determining CBF is the local energy demand of the tissue for cell maintenance, growth, differentiation, and myelination. Thus, significant increase in CBF observed between 8 and 17 days of age in rabbits occurs at a critical period of marked maturational advances in brain anatomy, connectivity, and behavior.

Whereas a rationale for increased CBF from 10 days of age to 25 days of age in normal rabbits has been described, the reason for the marked change in CBF and ICP from abnormally high on day 25 to normal on day 42 in rabbits with craniosynostosis is not well understood. With cerebral autoregulation, one may expect elevated ICP from craniosynostosis to result in either preserved CBF if the cerebral perfusion pressure (CPP) lies within the autoregulatory range or decreased CBF if the ICP is elevated to the extent that CPP is below the autoregulatory range. However, the CBF was actually greater in craniosynostosis rabbits than in normal rabbits at 25 days of age, which may represent an upward shift of the autoregulation curve. Shifts in the autoregulation curve have been described in humans and animals with orthostatic changes in sympathetic tone related to central venous pressure [40, 41]. Additionally, intracranial hypertension has been associated with defective autoregulation, and in a study of dogs with induced ICP elevations, hyperemia was often but not always seen with defective autoregulation [42]. Measuring ICP and CBF in WT and EOCS rabbits over a range of mean arterial blood pressure would help determine whether the observed changes in CBF are related to a change in position on the autoregulation curve, a shift of the curve, or a change in shape of the curve. Furthermore, shorter intervals between ICP and CBF measurements could help determine whether the return to normal CBF at 42 days precedes or follows the return to normal ICP.

Human data is sparse with regards to the influence of surgical correction on CBF. A Japanese group published their results on the use of I-123-IMP single photon emission computed tomography (SPECT) imaging studies in thirteen patients with craniosynostosis before and after surgical correction, with eight patients showing a focal area of hypoperfusion on the preoperative study [43]. Following surgery, there was improvement or resolution of the hypoperfusion in six of the eight patients. In another case series, Sen and colleagues (1995) reported on the use of technetium-99m HMPAO SPECT to study cerebral perfusion in seven children with craniosynostosis [44]. In all four patients who had pre- and postoperative studies done, cerebral hypoperfusion was detected by SPECT in the corresponding cerebral cortex underlying the affected suture in the preoperative scan, with an amelioration of the hypoperfusion following surgery. The cerebral hypoperfusion demonstrated in humans with craniosynostosis at various ages in development is in contrast to the hyperperfusion seen in rabbits with craniosynostosis at 25 days of age. The reason for this difference is unclear but may be related to different relative ages in development at which the cerebral blood flow measurements were made.

ASL-MRI represents a promising non-invasive imaging modality that could provide insight into the anatomical etiology of the neurodevelopmental deficits seen in patients with craniosynostosis. Furthermore, its ability to provide both quantitative and qualitative data with high spatial resolution represents key advantages over other imaging modalities. However, the reliability of the results of this study is limited by high variability from small sample sizes and the inability to test repeatability of CBF measures from ASL-MRI due to instrument decommissioning. Also, a comparison of CBF as measured by ASL-MRI in rabbits with unilateral coronal suture synostosis could add validity to the conclusion that CBF is abnormal in craniosynostosis if CBF were elevated in the same side of the brain as the fused suture.

While direct neurohistopathological analysis was beyond the scope of the present study, previous results from this rabbit model have shown that early suturectomy resulted in the preservation of white matter integrity as assessed by diffusion tensor imaging (DTI) [17], an increase in intracranial volume and a normalization of brain surface morphology as assessed with CT-imaging, and a normalization of cranial vault and cranial base angulation as assessed radiographically [13, 23, 24, 45, 46, 47]. Furthermore, studies have shown reossification of the suture following suturectomy can halt the initial improvements in intracranial volume and brain morphology after suturectomy, and further research has aimed to prevent reossification of the suture through molecular therapies [47–50]. In this model, direct neurohistopathological analysis is still needed to determine what effect the prevention of hyperemia through suturectomy has on neural growth and morphology.

Our well-established rabbit model of familial coronal suture synostosis [9–13, 17, 21, 25, 51] was used due to the similarity in the sutural, cranial vault, and cerebrovascular architecture dysmorphology, compared to infants with craniosynostosis [13, 21, 23, 39, 48–50, 52–54]; however, we acknowledge the limitation of extrapolating these results to a human clinical setting given the differences in brain morphology and growth patterns between humans and lagomorphs. Approximately 90% of brain growth in rabbits is completed by 35 days of age [13, 55], as compared to about 4 to 6 years of age in humans [56]. These findings suggest that the lagomorph neurocapsular matrix responds and adapts much more rapidly than the human and may be more exaggerated. Even with these limitations, this study demonstrates that early suturectomy prevents the superficial cortical hyperemia observed in response to intracranial hypertension seen in untreated animals.

Based on the result of this experiment alone, the influence of suturectomy on mitigating downstream functional deficits is unclear. Further studies with the rabbit model will have to be coupled with behavioral testing as well as histopathology. Ultimately, in the clinical setting, the use of imaging such as ASL-MRI, along with pre- and postoperative neuropsychological testing may provide further insight into the influence of surgical intervention in the neurocognitive development of children with craniosynostosis.

## Conclusion

Coronal suture synostosis is associated with aberrations of ICP and CBF in the developing brain. Using a well-established animal model of craniosynostosis and arterial spin-labeling MRI, CBF has been studied in the developing brain of rabbits with coronal suture synostosis. Previously, authors have demonstrated a two-fold increase in CBF in the superficial cortex at 25 days of age in EOCS rabbits compared to WT rabbits followed by a return to normal CBF at 42 days of age. The current study demonstrated that after coronal suturectomy in EOCS rabbits, the CBF at 25 days of age was similar to WT rabbits. These findings set the stage for further animal and human studies to determine whether there is a critical period during which suturectomy could minimize functional impairment secondary to craniosynostosis.

## Supporting information

S1 Table Dataset. Cerebral blood flow in mL/100g/min for each animal at each region of interest. Data for each age group are in successive tabs. The final tab shows mean cerebral blood flow in mL/100g/min for each group.
(XLSX)
